# Impact of three miniplate configurations on mental nerve integrity in parasymphyseal mandibular fractures: a blinded randomized trial

**DOI:** 10.1186/s12903-026-08487-0

**Published:** 2026-05-07

**Authors:** Reem M. Ismail, Ibrahim Mohamed Abdelhamed, Wafaa Samir, Amany M. Alryess, Yehia El-Mahallawy

**Affiliations:** 1 Oral and Maxillofacial Surgery Department, Faculty of Dentistry, Alexandria University, Champlion st, Azrite, Alexandria, Egypt; 2 Rheumatology and Rehabilitation, Faculty of Medicine, Alexandria University, Alexandria, Egypt

**Keywords:** Mandibular fractures, Parasymphysis, Mental nerve, Electrophysiological study

## Abstract

**Objectives:**

To assess the mental nerve’s functional integrity after parasymphyseal mandibular fracture fixation using three different miniplates configurations.

**Methodology:**

A total of 36 patients with unilateral parasymphyseal fractures were included in the study. Patients were randomly allocated into three groups based on the fixation modality configuration. Clinical evaluations were carried out in conjunction with radiographic assessments of mean bone density at the fracture line. Electrophysiological objective nerve testing was conducted 1 month postoperatively to assess the amplitude, onset latency, and conduction velocity of the mental nerve.

**Result:**

Physiological nerve testing intergroup analysis demonstrated no statistically significant differences in latency or amplitude, while conduction velocity showed a trend toward intergroup variation without reaching statistical significance (*P* = 0.072). Comparing the degree of agreement of nerve conduction parameters between the affected ipsilateral and healthy contralateral sides reported high levels of ICCs for all 3 miniplates configurations. Radiographic analysis revealed significantly greater bone density at the fracture site in the 3D-Interlocking group compared with twin fork and conventional miniplates (*P* < 0.001).

**Conclusion:**

Fixation of parasymphyseal mandibular fractures using 3D-Interlocking or Twin-Fork miniplates yielded a slightly enhanced mental nerve conduction profile and integrity preservation when compared to the conventional miniplate configuration. Furthermore, the 3D-Interlocking plate demonstrated improved stability and healing of the fracture line. These findings support the use of 3D-Interlocking or Twin-Fork miniplates as preferable alternatives to conventional miniplates in the mental foramen region.

**Trial Registration:**

Trial was retrospectively registered at clinicaltrials.gov [NCT07058597/ 2025-07-01].

**Supplementary Information:**

The online version contains supplementary material available at 10.1186/s12903-026-08487-0.

## Introduction

One of the most common types of traumatic injuries identified in the maxillofacial area is mandibular fractures. establishing a high percentage of facial skeletal injuries worldwide. Fractures in the mental nerve zone are among the prevailing mandibular fractures, which are typically accompanied by functional abnormalities and neurosensory complications [1, 2].

Fractures of the mandibular parasymphysis present unique challenges due to the proximity of the mental foramen and neurovascular bundle, where injury to the mental nerve can significantly impact a patient’s quality of life. Conventional two-miniplate fixation remains widely used, yet newer designs such as three-dimensional interlocking and twin-fork miniplates have been introduced to enhance stability while minimizing the risk of nerve compromise [3, 4]. However, no randomized controlled trial has yet to compare the effect of three miniplate configurations on mental nerve function or bone healing in parasymphyseal fractures.

The primary aim of this study was to compare the neurosensory integrity of the mental nerve and bone healing following fixation of parasymphyseal mandibular fractures using three different miniplate configurations, under the null hypothesis that no significant differences would exist in electrophysiological or bone density outcomes among the fixation modalities. The secondary aim was to evaluate and compare the clinical performance of the three systems in the management of parasymphyseal fractures through a randomized prospective trial design.

## Materials and methods

### Study design

**Fig. 1:**
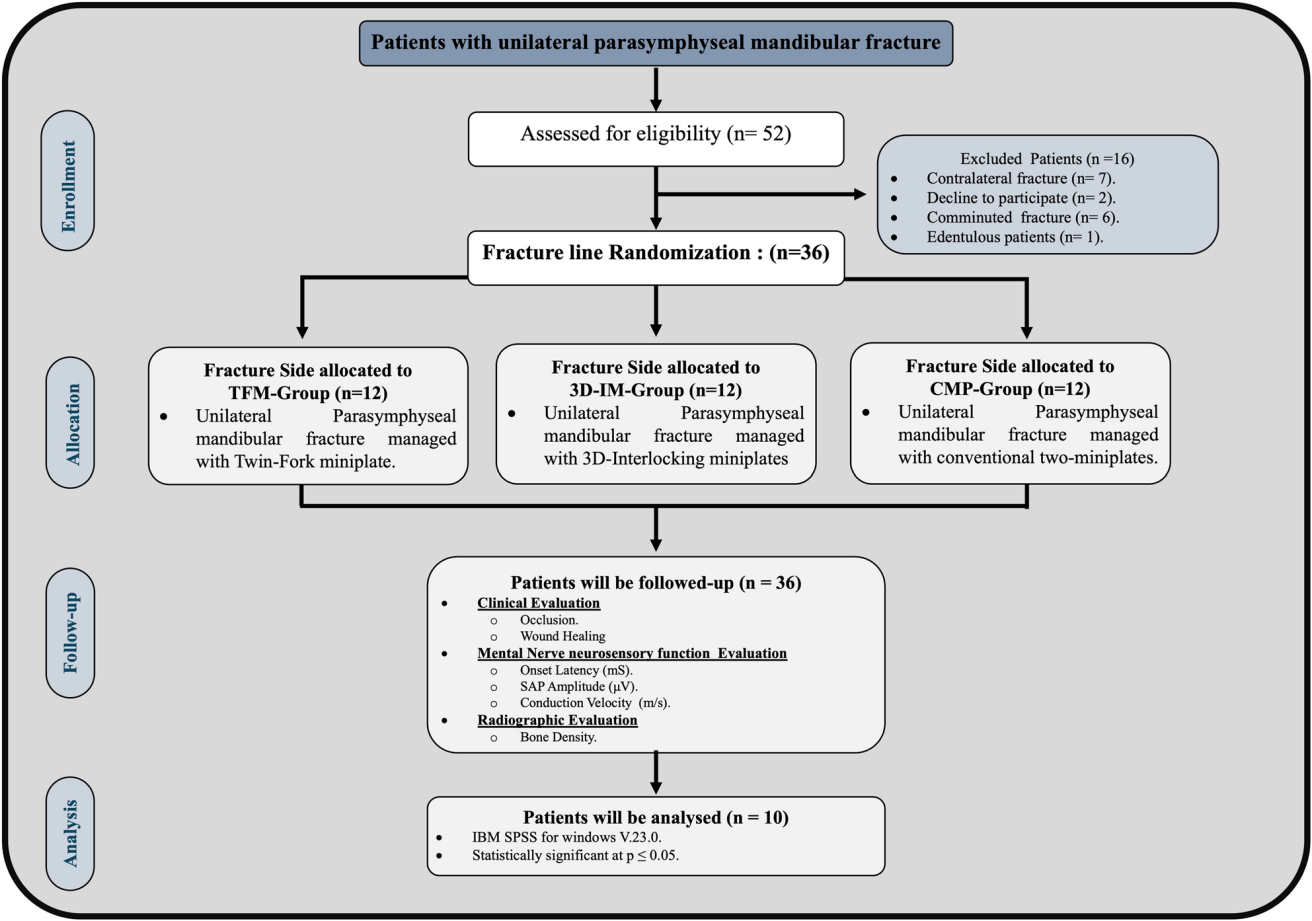
CONSORT Flow chart

This randomized controlled trial, adhering to CONSORT guidelines (http://www.consort-statement.org), compared Twin-Fork, 3D-Interlocking, and conventional double configuration miniplates for fixation of mandibular parasymphyseal fractures (Fig. 1). Sample size was estimated, assuming 5% alpha error and 80% study power. According to Song Q et al. [5], the percentage of patients with worsened neurosensory scores was 74% for those who received two miniplates, compared to 26% for those with one miniplate. The minimum required sample size was calculated to be 11 patients per group, increased by 10% to 12 to make up for loss to follow-up. The total required cohort sample size is 36 patients. Sample size was based on Rosner’s method, calculated by Gpower 3.0.10 [6, 7].

### Patients’ selection

The study included patients suffering from recent unilateral parasymphyseal mandibular fractures requiring open reduction and internal fixation from August 2020 to March 2025. The fracture line must be distal to the anterior transition zone and require double miniplate fixation as per Champy’s recommendation. Adults without gender predilection who agreed to follow-up and are medically fit for general anesthesia were included in this study. Exclusion criteria included bilateral fractures in the mental nerve zone, fractures with infection, pathological or old fractures, completely edentulous patients, and comminuted fractures. Fracture lines in the body of the mandible were also excluded from the study. Patients were allocated in a 1:1:1 ratio using an on-site computer software system with concealed allocation through sequentially numbered, opaque, sealed envelopes (SNOSE). Randomization was conducted with 3, 6 & 9 random block sizes (http://www.randomizer.org/). Patients were randomly allocated into either the *TFM Group*, treated with Twin-Fork miniplate, *3D-ILMP Group*, treated with 3D-Interlocking miniplates, or *CMP Group*, treated with conventional two-miniplates. Surgeries were conducted at Alexandria University’s Oral and Maxillofacial Surgery Department under Helsinki guidelines with ethical approval (IRB:0156-09/2020), and trial registration (ClinicalTrials.gov/ NCT07058597).

The Twin-Fork miniplate system is manufactured using a medical-grade 5 titanium alloy with a profile of 1.5 mm [8]. It has an upper horizontal arm serving as a tension band and a lower horizontal arm acting as the compression arm. Both arms are connected to a single horizontal arm, which coincides with the Osteosynthesis lines distal to the region of the mental foramen (Supplementary Fig. 1) [8]. The 3D-interlocking miniplate system consists of two parts: a U-shaped plate and a linear plate, each with a thickness of 1.5 mm. The superior margins of the tissue-contacting surface of the U-shaped plate contain circular recesses where the thickness is reduced to 0.75 mm. Similarly, the lateral ends of the linear plate have corresponding recesses of the same thickness, allowing both plates to interlock securely. At the interlocking sites, the combined thickness returns to 1.5 mm, consistent with the rest of the plate. The vertical strut of the U-shaped plate is designed in varying lengths, ranging from 6 to 9 mm [4]. The 2.0 mm miniplates system is provided with a 1.5 mm Plate profile. The supplier of the three mini-plate systems was standardized, and 2.0-mono-cortical screws were utilized for plate fixation in all three groups (Arab Engineer’s Co, Cairo, Egypt). (Supplementary Fig. 2).

### Preoperative assessment

A comprehensive history taking and methodical clinical examination were implemented and logged for all of the enrolled cases. Subjective assessment of the affection to the mental nerve sensation was conducted for both sides of the face. A Computed Tomography (CT) scan was conducted to determine the number and pattern of fracture lines, displacement severity, and the presence of teeth within the fracture line (Philips Brilliance 64 MDCT, Philips, Eindhoven, Netherlands).

### Surgical procedure

All patients underwent the required preoperative investigations to obtain an anesthesiologist’s clearance and were instructed to fast for 8 h before surgery. Surgical treatment was conducted under general anesthesia using nasotracheal intubation. The surgical field was prepared with povidone-iodine scrub solution, followed by sterile draping to expose only the operative site.

Access to the fracture line was achieved through an intraoral vestibular approach, which was conducted superiorly to the preoperatively radiographically detected mental nerve foramen, all while maintaining a minimum of 5-mm keratinized mucosal cuff. This was pursued by mobilization of the fracture with careful removal of any soft tissue interposed within the fracture line. Teeth involved in the fracture were either preserved or extracted as indicated. Surgical skeletonization of the mental nerve was carefully conducted through a series of dissecting the periosteal envelope and blunt dissection in a vector parallel to the nerve. This permitted an ample mucoperiosteal flap retraction whilst preserving the integrity of the nerve bundle. Anatomical reduction of the fracture was accomplished to restore proper occlusion, aided by temporary Intermaxillary Fixation (IMF) as needed to guide the reduction. The surgical team was unchanged for all of the patients in this study (R.I & I.A). Before selecting the fixation modality, an assigned personnel member was entrusted with opening the sealed envelope for group allocation (Y.E). This allowed allocation concealment and a standardized nerve handling and skeletonization approach in all 3 groups.

**Fig. 2:**
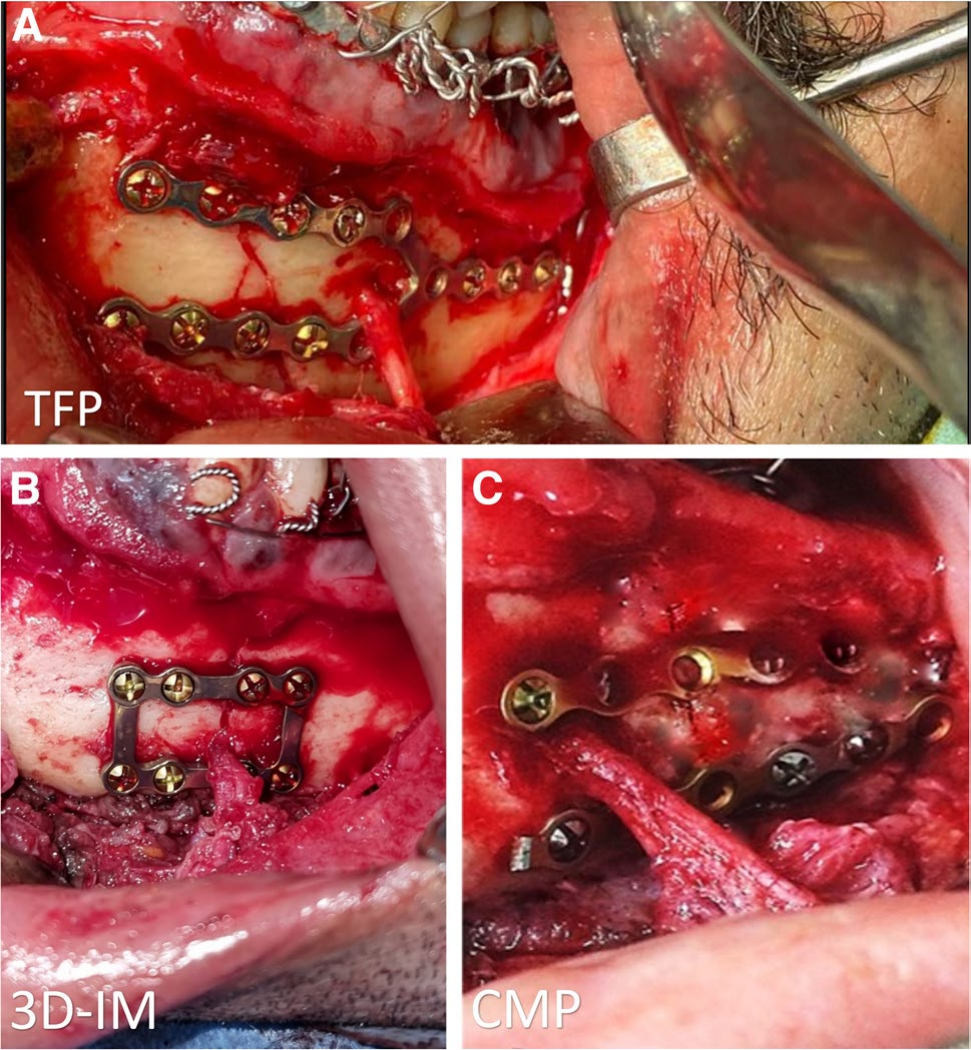
Clinical picture of fracture fixation. **A**, Twin-Fork miniplate. **B**, 3D-interlocking miniplate. **C**, Conventional double miniplate

For patients in the *TFM Group*, the twin-fork miniplate was positioned across the fracture line, and 2.0-mono-cortical screws were applied strategically to avoid injury to the roots of preserved teeth, with lengths ranging from 5 to 7 mm (Fig. 2A). For those in the *3D-ILMP Group*, the U-shaped segment of the 3D-Interlocking miniplate was positioned around the fracture line, ensuring vertical bars aligned parallel to the fracture. Fixation commenced with 7 mm screws placed at the inferior border on each side of the fracture. Subsequently, the linear (horizontal) plate was aligned in the subapical region, interlocked with the U-shaped plate via designed recesses, and secured using 7-mm screws at its upper border (Fig. 2B). For patients in the control *CMP Group*, 2 traditional miniplates were applied along Champy’s lines across the fracture site and fixed using mono-cortical screws with respect to the position of the roots (Fig. 2C). Upon completion of fixation in all groups, the IMF was released, and occlusion was thoroughly assessed. The surgical wound was then closed meticulously to promote optimal healing and minimize postoperative complications.

Following surgery, patients were instructed to apply an ice pack extra-orally for the first 24 h to minimize swelling, and a soft, high-protein, high-calorie diet was recommended for four weeks postoperatively. A five-day course of 875-mg amoxicillin + 125-mg clavulanic acid was prescribed, along with 500-mg Metronidazole. α-chemo-trypsin ampoules and anti-edematous therapy, alongside 50 mg diclofenac potassium analgesic, were both prescribed for a 5-day duration.

### Clinical follow-up phase

Clinical outcomes were evaluated according to a structured follow-up schedule at 24 h, 1, 4, and 6 weeks postoperatively using standardized indices. Clinical follow-up was conducted by an assigned personnel, blinded from the surgical procedure and group allocation procedure (A.A). Occlusal evaluation was performed by examining the maximum intercuspation position. Particular attention was given to molar and canine interdigitation as well as midline centralization. Any occlusal discrepancies—including open bite, midline shifts, or inappropriate tooth contacts—were carefully identified and documented. The surgical site was examined for clinical signs of infection, wound dehiscence, or other complications.

### Mental nerve function assessment

Mental nerve sensory function assessment was conducted using the nociceptive electrophysiological method one month postoperatively. A standardized dental probe was applied with gentle pressure along the distribution area of the mental nerve. Findings were compared to the contralateral (unaffected) side to identify areas of altered sensation, anesthesia, or paresthesia. The electrophysiological evaluation was performed to assess sensory conduction, Nerve Conduction Velocity (NCV), and Sensory Action Potential (SAP) of the mental Nerve (IAN). Testing was conducted using the Neuropack Two electromyograph apparatus (MEB-9400), following the protocol described by Jaaskelainen et al. [9]. Surface electrodes were used to record the mental nerve conduction orthodromically. The active recording electrodes were positioned approximately 3 cm anterior to the tragus, beneath the zygomatic arch, in front of the temporomandibular joint. The reference electrode was placed on the skin just above the zygomatic arch, while the ground electrode was attached around the patient’s upper arm. Stimulation was applied using a bipolar surface-stimulating electrode. The cathode was positioned on the mental nerve at its exit from the mental foramen, with its location estimated by palpation at the premolar region. Electrical stimuli consisted of 0.2-ms square-wave pulses at intensities three to five times the sensory threshold (6–15 mA). The electrophysiological nerve testing was conducted by an experienced Physical Medicine & Rehabilitation (PM&R) physician, who was blinded regarding group allocation (W.E). The mental nerve sensory Onset Latency, in milliseconds (ms), SAP Amplitude, in microvolts (µV), and nerve Conduction Velocity (NCV), in meters per second (m/s), were measured and reported in the fractured side and compared with the healthy contralateral side in all the enrolled patients in this study [8, 9]. For each of the nerve conduction parameters, the percentage of difference between the affected ipsilateral and the healthy contralateral sides was calculated. The following equation was utilized: [(𝑥̄_Affected_- 𝑥̄_Healthy_) / 𝑥̄_Healthy_] x 100 [8, 9].

### Radiographic evaluation and bone density assessment

**Fig. 3:**
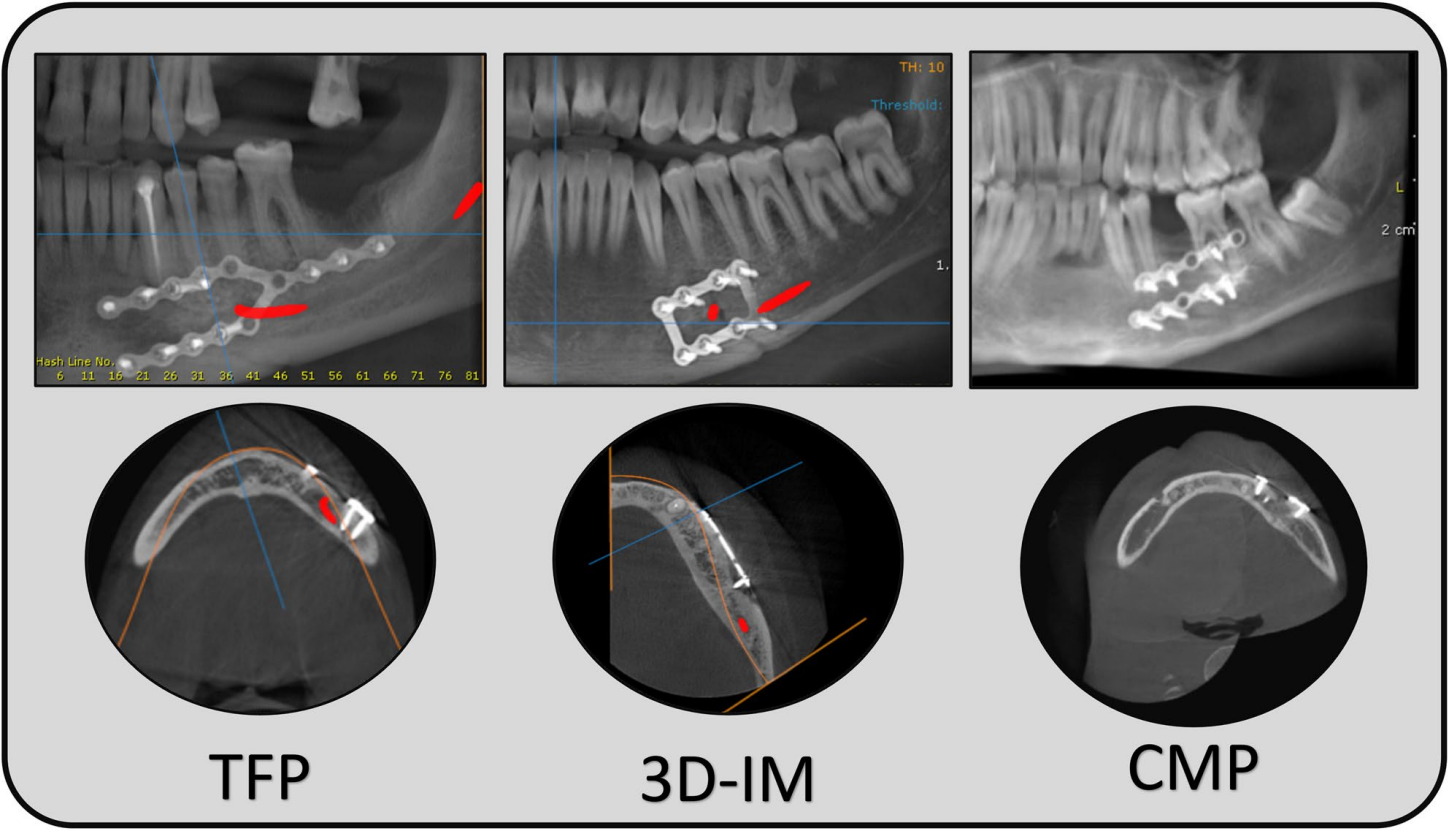
Postoperative radiographic assessment for the three miniplates configurations. Bone density analysis was conducted by the application of six 3_ × _3 regions of interest along the fracture line, where the mean value for each record was calculated

An immediate Cone-Beam Computed Tomography (CBCT) scan was performed to evaluate the adequacy of fracture reduction and fixation, followed by a second scan at the 12th postoperative week. Both scans were obtained at the institution’s radiographic center, with a standardized machine, phantom calibration, and acquisition parameters. Assessment of the mean bone density at the fracture line was performed at the 12-week scan compared to the initial postoperative scan. Bone mineral density was measured on CBCT using OnDemand software (OnDemand 3D APP-DBM, Cybermed, Seoul, South Korea). Six measurements were taken along the fracture line, and the mean value was calculated for each patient [10]. Bone density values were expressed in Hounsfield Units (HU) after conversion from grayscale values [11] (Fig. 3).

### Statistical analysis

Data were fed to the computer and analyzed using the IBM SPSS software package version 20.0. (Armonk, NY: IBM Corp, released 2011). The Shapiro-Wilk test was used to verify the data’s normality of distribution, and the significance was judged at the 5% cutoff value. The Chi-square test for categorical variables, with the Monte Carlo correction when more than 20% of the cells have expected count less than 5. The F-test (ANOVA) was utilized for normally distributed quantitative variables to compare more than two groups, with the Tukey Post Hoc test for pairwise comparisons. The Paired t-test was utilized to compare two periods. Neurosensory functional agreement between the fracture-affected side and the contralateral side was analyzed using a two-tailed Intra Class Correlation Coefficient test (ICC) [12]. A key to apprehending the ICC outcome is presented in the supplementary data.

## Results

This study included 36 patients diagnosed with parasymphyseal mandibular fractures, divided equally into three groups. The demographic analysis revealed a male predominance, with a male-to-female ratio of 2.27:1, with an average age of 29.50 ± 7.79 years. Road traffic accidents were identified as the leading cause of injury, accounting for 72.2% of cases (*n* = 26), while the remaining cases (27.8%, *n* = 10) were attributed to falls and interpersonal violence (Table 1).

**Table 1:** Demographic data and epidemiology of the study (*n* = 36)

*n* = 36	No. (%)
Gender	
Male.	25 (69.4%)
Female.	11 (30.6%)
**Age (years)**	
Min. – Max.	18.0–48.0
Mean ± SD.	29.50 ± 7.79
**EOT**	
RTA.	26 (72.2%)
Claimed Falls.	5 (13.9%)
IPV.	5 (13.9%)

*EOT *Etiology of Trauma, *RTA *Road Traffic Accident, *IPV *Inter Personal Violence

Occlusal assessment revealed proper intercuspal and canine relationships in all patients across the three groups, with no need for selective grinding during the follow-up period. Interfragmentary mobility at the fracture site was evaluated, and slight mobility was observed in only one patient (8.3%) from the CMP group during the first postoperative week; this resolved by the fourth week. No mobility was detected in patients from the 3D-ILMP or TFM groups. However, the intergroup difference was not statistically significant (*P* = 1.000). In terms of wound healing, delayed wound closure occurred in one patient (8.3%) in both the 3D-ILMP and TFM groups. In the CMP group, two patients (16.7%) exhibited wound dehiscence. All wounds had completely healed by the sixth postoperative week across all groups.

All three groups exhibited a statistically significant increase in mean bone density on the 3-month postoperative CBCT scans when compared to the immediate postoperative measurements (*P* < 0.001^*^). While no significant intergroup differences were observed in the immediate postoperative bone density values (*P* = 0.380), the 3D-ILMP group showed a significantly higher mean bone density at 3 months (1096.4 ± 74.40 HU) compared to the CMP (968.0 ± 70.41 HU) and the TFM groups (954.7 ± 62.08 HU) (*P* < 0.001^*^) (Table 2).

**Table 2:** Bone Density (Hu) analysis for the three miniplates configurations

*Bone Density (Hu) *(***n* = 36)**		TFP (*n* = 12)	3D-ILMP (*n* = 12)	CMP (*n* = 12)	F (*p*)
**Baseline Immediate Postoperative Scan**	Mean ± SD.	656.7 ± 83.3	695.3 ± 80.9	660.0 ± 55.3	0.996 (0.380)
	Median.	656.7	695.3	657.5	
	Min – Max.	529.7–783.7	571.9–818.6	584.0–747.4	
**3-month’ Postoperative Scan**	Mean ± SD.	954.7 ± 62.1	1096.4 ± 74.4	968.0 ± 70.4	15.363* (0.001*)
	Median.	954.7	1107.4	960.9	
	Min – Max.	860.0–1049.4	956.2–1196.5	871.3–1103.9	
***p***		**< 0.001** ^*^	**< 0.001** ^*^	**< 0.001** ^*^	

*Hu *Hounsfield Units, *TFP *Twin-Fork Miniplates, *3D-IMP *3D-Interlocking Miniplates, *CMP *Conventional Miniplates, *Fr *F for One way ANOVA test, *SD * Standard deviation; *Statistically significant difference at *P*-value < 0.05

Physiological Nerve Testing intergroup analysis of the mental nerve conduction parameters reported a statistically insignificant difference. (***P***_Latency_=0.288, ***P***_CV_=0.072, ***P***_Amplitude_=0.564) (Table 3).

**Table 3:** Intergroup objective physiological nerve testing analysis for the three miniplates configurations

Physiological nerve testing (Affected Side)		Latency (ms)	Conduction Velocity (m/s)	Amplitude (µV)
**TFP (** ***n*** ** = 12)**	Mean ± SD.	3.83 ± 1.05	28.98 ± 11.47	8.11 ± 1.90
	Min. – Max.	2.02–4.90	19.40–47.20	5.0–11.0
**3D-ILMP (** ***n*** ** = 12)**	Mean ± SD.	4.31 ± 0.38	37.30 ± 9.32	8.38 ± 2.52
	Min. – Max.	3.80–5.0	22.90–49.30	5.20–14.30
**CMP (** ***n*** ** = 12)**	Mean ± SD.	3.98 ± 0.66	37.53 ± 8.93	7.48 ± 1.85
	Min. – Max.	2.90–4.70	26.50–49.60	5.10–11.0
**F ** **(** ***P*** **)**		1.292 (0.288)	2.860 (0.072)	0.583 (0.564)

*MS *Milliseconds, *m/s *meter per second, *µV *microvolts, *TFP *Twin-Fork Miniplates, *3D-ILMP *3D-Interlocking Miniplates, *CMP *Conventional Miniplates, *Fr *F for One way ANOVA test, *SD *Standard deviation;*Statistically significant difference at *P*-value < 0.05

The percentage of difference (% of Δ) between the affected ipsilateral and the healthy contralateral sides was calculated for all of the nerve conduction parameters, which reported measurable differences between healthy and affected sides across all fixation systems. Latency was consistently prolonged postoperatively, with the greatest percentage of increase in the TFM group (_**+**_25.85%), followed by the 3D-ILMP (_**+**_23.04%) and CMP (_**+**_17.22%) groups. Conduction velocity showed the largest percentage of decrease in the TFP group (**-**18.38%), compared to the 3D-ILMP (**-**7.99%) and CMP (**-**3.66%) groups, suggesting relatively better preservation of nerve conduction in the latter two systems. Amplitude reductions were mild across all groups, with the smallest decrease observed in the 3D-ILMP group (**-**5.43%), followed by TFP (**-**6.30%) and CMP (**-**9.83%) groups. This could be indicative of the extent of axonal damage in the CMP group (Table 4).

**Table 4:** Physiological nerve testing analysis of the degree of agreement between the fractured ipsilateral and healthy contralateral sides within each miniplate configuration (*n* = 36)

Healthy vs. Affected	Latency (ms)				Conduction Velocity (m/s)				Amplitude (µV)		
	TFP	3D-ILMP	CMP		TFP	3D- ILMP	CMP		TFP	3D- ILMP	CMP
**% of Δ**	_(+)_25.85	_(+)_23.04	_(+)_17.22		_(−)_18.38	_(−)_7.99	_(−)_3.66		_(−)_6.30	_(−)_5.43	_(−)_9.83
**ICC**	0.866	0.700	**0.968**		0.863	**0.982**	**0.995**		**0.973**	**0.991**	**0.979**
**95% C.I**	(0.60– 0.96)	(0.24– 0.90)	(0.89–0.99)		(0.59– 0.96)	(0.94– 0.99)	(0.98–0.99)		(0.91–0.99)	(0.97–0.99)	(0.93–0.99)
***P***	< 0.001^*^	0.004^*^	< 0.001^*^		< 0.001^*^	< 0.001^*^	< 0.001^*^		< 0.001^*^	< 0.001^*^	< 0.001^*^

*MS *Milliseconds, *m/s *meter per second, *μV *microvolts, *TFP *Twin-Fork Miniplates, *3D-ILMP *3D-Interlocking Miniplates, *CMP *Conventional Miniplates; *% of Δ *Percentage of difference between Affected and Healthy sides, *(+) *Increase in mean value in the affected side, *(-) *Decrease in mean value in the affected side, *ICC *Intra class Correlation coefficient, *C.I *Confendance Interval ;*Statistically significant difference at *P*-value <0.05

ICC Outcome Values: <0.5 Poor agreement, 0.5 to <0.75 Moderate agreement, 0.75 to <0.9 Good agreement, 0.9 - 1.0 Excellent agreement

All fixation configurations showed consistently high levels of ICCs agreement between the healthy and affected sides for the investigated nerve conduction parameters. In terms of latency, the 3D-ILMP group showed acceptable-moderate agreement (ICC = 0.700), while the TFM group showed excellent agreement (ICC = 0.968), and the CMP group showed good agreement (ICC = 0.866). Regarding conduction velocity, the CMP group maintained good agreement (ICC = 0.863), whereas the 3D-ILMP (ICC = 0.982) and TFM systems (ICC = 0.995) both achieved excellent agreement. All three fixation methods demonstrated excellent agreement for amplitude measurements (**ICC**_TFM_ = 0.973, **ICC**_3D−ILMP_=0.991, **ICC**_CMP_ = 0.979) (Table 4).

## Discussion

Surgeons are still at odds on how to treat mandibular fractures, especially those that affect the area where the body and parasymphysis meet. The biomechanical complexity of this transition zone is attributed to the different patterns of transmitted functional stress and force during speech and mastication [13]. Furthermore, additional anatomical complexity of the anterior transition zone emerges in its close anatomical relationship with the mental nerve [3]. The fracture’s detrimental effect, along with the required nerve mobilization, skeletonization, and retraction for hardware placement, inflicts a significant chance of neurosensory disruption [14]. The goal of this study was to assess mental nerve neurosensory integrity following fracture fixation in the mandibular parasymphyseal area with three different miniplate configurations.

The study included 36 patients, with 69.4% males and 30.6% females. The participants’ ages ranged from 18 to 48 years, with a mean age of 29.50 ± 7.79 years. Regarding the mode of trauma, road traffic accidents were the predominant cause, accounting for 72.2%, followed by interpersonal violence and stated falls, each responsible for 13.9%. These findings could be elucidated by the fact that men tend to engage in risky behaviors, outdoor activities, and occupations that put them at greater peril and risk of trauma. Men are more likely to be involved in accidents because they tend to drive aggressively and drive cars or motorcycles more frequently. Owing to social and cultural norms, men are more likely to engage in violence and interpersonal conflicts [15–17].

In the current analysis, all three fixation technique configurations reported gradual improvement in postoperative occlusion over time, with all patients achieving total stability by the sixth postoperative week. This is consistent with prior research promoting the effectiveness of the functionally stabilized fixation concept, where the placement of miniplates along the lines of osteosynthesis provides effective functional management of the mandibular fracture line [3, 18].

Even though there were some slight differences in the stability of the CMP group at 24 h and the first week following surgery, these differences were not statistically significant and did not affect the final clinical outcome. Earlier comparative studies have shown similar trends, with plate configuration influencing initial rigidity but having little effect on long-term stability once proper reduction and fixation were achieved [19].

Wound dehiscence was detected in 8.3% of patients in the TFM and 3D-ILMP groups and in 16.7% of patients in the CMP group, indicating that the complications associated with wound healing were minimal. By the sixth week, every case had been resolved without the need for additional assistance. Liu et al. reported that the incidence of postoperative complications such as wound dehiscence, malocclusion, and hardware failure was significantly lower with three-dimensional plates compared to conventional miniplates [2]. Overall, the results indicate that all three techniques can produce favorable clinical performance, with excellent occlusal outcomes and full healing in six weeks, even though early stability may differ slightly based on the fixation scheme. Therefore, rather than depending only on variations in short-term stability, the choice of fixation technique should take into account factors like fracture pattern, surgeon preference, and anatomical constraints. A deeper understanding of the variations in performance between these fixation systems may be possible with future research involving larger cohorts and biomechanical analysis.

Three months after surgery, the progressive increase in bone density observed across all fixation groups reflects the normal course of bone remodeling and fracture healing following stable fixation. All groups showed statistically significant improvement from the immediate to 12-week postoperative scan values (*P* < 0.001), indicating that fixation is sufficient in establishing a favorable biological environment for bone consolidation. Although initial postoperative bone density values did not significantly differ among groups (*P* = 0.380), the 3D-ILMP group had a significantly higher mean bone density at three months (1096.4 ± 74.40 HU) than both the two-CMP (968.0 ± 70.41 HU) and the TFM groups (954.7 ± 62.08 HU). According to this, the 3D-ILMP design may contribute to a higher mechanical stability, which would minimize micro-movements at the fracture site and improve osteogenesis, which is consistent with previous studies that demonstrated the favorable effect of 3D-miniplates on bone healing promotion [20, 21]. Although the OnDemand3D workflow includes HU readouts and is commonly utilized therapeutically, absolute CBCT-HU values are not consistent between devices and acquisitions. The best technique is to analyze within-patient, within-protocol changes rather than comparing absolute HU to other datasets [22].

Physiological nerve testing intergroup analysis demonstrated no statistically significant differences in latency or amplitude, while conduction velocity showed a trend toward intergroup variation without reaching statistical significance. These findings indicate a degree of transient postoperative neurosensory impairment, with modest differences in nerve conduction parameters that were, in many cases, not statistically significant between groups. However, 3D-ILMP and TFM groups demonstrated somewhat better conduction profiles compared to CMP, as evidenced by comparatively smaller reductions in conduction amplitude and velocity. These patterns might be linked to variations in biomechanical behavior, which could lead to less manipulation of the mental nerve during surgery or compression of it afterward. These findings were supported by ICC analysis, which showed good conduction velocity and amplitude agreement between the healthy and affected sides in the 3D-ILMP and TFM groups, indicating a relative preservation of nerve function. Therefore, 3D-ILMP and TFM might be promising substitutes for traditional miniplates; however, before definitive clinical recommendations can be made, larger studies with longer follow-up are needed.

The postoperative reductions seen in our study can be deciphered in light of Deeb et al., who reported a mean amplitude of 9.9 ± 5.5 µV in healthy individuals [23]. Politis et al. noted that transient edema or ischemia caused by manipulation of the soft tissues surrounding the mental nerve may contribute to early postoperative sensory changes [24]. Electrophysiological testing in our study was performed one month after surgery to minimize these confounding factors. Long-term recovery trends reported in the literature support our findings. Cillo et al. suggested that functional sensation of the mental nerve is typically restored within 2–3 months following surgical skeletonization [14].

Collectively, these observations indicate that although transient postoperative neurosensory disturbances are common, the 3D-ILMP and TFM configurations may offer biomechanical advantages that reduce intraoperative manipulation and postoperative compression of the mental nerve, thereby facilitating earlier and more complete recovery when compared to the conventional fixation pattern.

Intra-class correlation coefficients (ICCs) across latency, conduction velocity, and amplitude physiological parameters showed high levels of agreement between the healthy and affected sides for all fixation configurations. In terms of latency, the TFM group had excellent agreement (ICC = 0.968), the CMP group had good agreement (ICC = 0.866), and the 3D-ILMP group had adequate moderate agreement (ICC = 0.700). Both 3D-ILMP (ICC = 0.982) and TFM (ICC = 0.995) showed excellent agreement in terms of conduction velocity, while the CMP group showed good agreement (ICC = 0.863). All three fixation systems showed excellent agreement in amplitude measurements, with ICC values greater than 0.97.

These results show that all three fixation techniques maintained similar physiological nerve function, even with minor differences in latency and conduction velocity agreement levels. The comparable outcome in the three groups could also be attributed to the standardization of the surgical team and group allocation after full fracture exposure. This ensured an equal and random amount of skeletonization for all of the cases, regardless of the utilized miniplate configuration. The moderate latency agreement and good conduction velocity outcome of the 3D-ILMP group could be attributed to the increased retraction and focal compression on the nerve, which is evident during the application of the strut design of the 3D-plate.

In the present study, fixation with 3D-ILMP was slightly more difficult and required more time compared to both CMP and TFM patterns. This may be attributed to the additional caution exercised by the surgeon in preserving the mental nerve while adapting and securing the U-shaped part across the neurovascular bundle. Extra care was also required during drilling for the TFM, given its relatively longer design and the higher probability of injuring the roots of adjacent teeth. Similar caution was practiced during fixation of the superior plate in the CMP group to avoid root injury. In contrast, the 3D-ILMP group rarely encountered this concern, as the plate design required fewer screws of shorter length for fixation. These surgical impressions are subjective and may evolve as the learning curve improves with execution familiarization.

Important considerations concerning the best internal fixation approach are brought up by the dynamic nature of these biomechanical forces. The surgical strategy must therefore strike a balance between the necessity of maintaining nerve function and the requirement for efficient stabilization, necessitating careful technique and a deep comprehension of regional anatomy.

The single-center nature of this report could act as a limiting factor for the generalizability of the study outcomes. Furthermore, the short follow-up for the neurosensory analysis could not appraise the neural re-innervation and chronic neurosensory recovery. This, along with the modest recruited cohort, could call for the need for a retrospective appraisal of the long-term effects of the different fixation modalities. The retrospective trial registration could also be pointed out as a limitation of the study. Furthermore, the study used Hounsfield unit (HU) values derived from CBCT, which are known to be affected by acquisition protocol and device-specific variability. HU analyses were limited to intra-patient comparisons made with the same CBCT device and imaging protocol in order to lessen this restriction and highlight relative rather than absolute changes. Blinding of the neurosensory outcome assessor was important in evading expectation bias. This could not be performed with either the operator or the radiographic assessor.

## Conclusion

All three fixation techniques proved to be clinically effective in the management of parasymphyseal mandibular fractures, with comparable outcomes in occlusal stability, fracture healing, and neurosensory preservation. Although minor differences were observed, the 3D-ILMP miniplates demonstrated superior postoperative bone density, while the twin-fork and 3D-ILMP designs showed relative preservation of nerve conduction compared to the two-miniplate system.

## Supplementary Information

Supplementary Material 1: Supplementary Table 1. A key to apprehending the outcome values of the ICC values

Supplementary Material 2: Supplementary Table 2. Occlusion analysis for the three miniplates configurations

Supplementary Material 3: Supplementary Figure 1. Photographic Illustration of the Twin-Fork miniplate

Supplementary Material 4: Supplementary Figure 2. Illustration of the three miniplates configurations utilized in this study

## Data Availability

All data generated or analysed during this study are de-identified and available for whom included in this published article.

## Abbreviations

TFM: Twin-Fork miniplate
CMP: Conventional miniplate
SNOSE: Sequentially numbered, opaque, sealed envelopes
IMF: Intermaxillary Fixation
SAP: Sensory Action Potential
μV: microvolts
HU: Hounsfield Units
3D-ILMP: 3D-Interlocking miniplates
CT: Computed Tomography
PM&R: Physical Medicine & Rehabilitation
NCV: Conduction Velocity
ms: milliseconds
m/s: meters per second
CBCT: Cone-Beam Computed Tomography
