# Supplementary material for: Impact of three miniplate configurations on mental nerve integrity in parasymphyseal mandibular fractures: a blinded randomized trial
Source: BMC Oral Health. 2026 May 7;26:859. doi: 10.1186/s12903-026-08487-0 (PMC13173844; doi:10.1186/s12903-026-08487-0)
Supplement: Supplementary file 1 — Supplementary Material 1: Supplementary Table 1. A key to apprehending the outcome values of the ICC values [file 12903_2026_8487_MOESM1_ESM.docx]

| ***Supplementary Table 2: Occlusion analysis for the three miniplates configurations.*** | | | | |
| --- | --- | --- | --- | --- |
| ***Occlusion Analysis*** | **TFP (n=12)** | **3D-IM (n=12)** | **CMP (n=12)** | **Fr**  **(*P*)** |
|  | | | | |
| **24h Post-operative** |  |  |  |  |
| Stable | 8 (66.7%) | 8 (66.7%) | 7 (58.3%) | 2.767  (0.965) |
| Mildly unstable | 1 (8.3%) | 0 (0.0%) | 2 (16.7%) |  |
| Moderately unstable | 2 (16.7%) | 3 (25.0%) | 2 (16.7%) |  |
| Severely unstable | 1 (8.3%) | 1 (8.3%) | 1 (8.3%) |  |
|  | | | | |
|  | | | | |
| **1^st^ week** |  |  |  |  |
| Stable | 8 (66.7%) | 9 (75.0%) | 8 (66.7%) | 1.172  (1.000) |
| Mildly unstable | 3 (25.0%) | 2 (16.7%) | 2 (16.7%) |  |
| Moderately unstable | 1 (8.3%) | 1 (8.3%) | 2 (16.7%) |  |
| Severely unstable | 0 (0.0%) | 0 (0.0%) | 0 (0.0%) |  |
|  | | | | |
|  | | | | |
| **4^th^ week** |  |  |  |  |
| Stable | 11 (91.7%) | 12 (100%) | 10 (83.3%) | 1.995  (0.756) |
| Mildly unstable | 1 (8.3%) | 0 (0.0%) | 2 (16.7%) |  |
| Moderately unstable | 0 (0.0%) | 0 (0.0%) | 0 (0.0%) |  |
| Severely unstable | 0 (0.0%) | 0 (0.0%) | 0 (0.0%) |  |
|  | | | | |
|  | | | | |
| **6^th^ week** |  |  |  |  |
| Stable | 12 (100%) | 12 (100%) | 12 (100%) | –  (-) |
| Mildly unstable | 0 (0.0%) | 0 (0.0%) | 0 (0.0%) |  |
| Moderately unstable | 0 (0.0%) | 0 (0.0%) | 0 (0.0%) |  |
| Severely unstable | 0 (0.0%) | 0 (0.0%) | 0 (0.0%) |  |
|  | | | | |
| **TFP**: Twin-Fork Miniplates; **3D-IMP**: 3D-Interlocking Miniplates; **CMP**: Conventional Miniplates; **χ2**: Chi square test; **MC**: Monte Carlo;*****Statistically significant difference at *P*-value <0.05. | | | | |
