# Supplementary material for: Impact of three miniplate configurations on mental nerve integrity in parasymphyseal mandibular fractures: a blinded randomized trial
Source: BMC Oral Health. 2026 May 7;26:859. doi: 10.1186/s12903-026-08487-0 (PMC13173844; doi:10.1186/s12903-026-08487-0)
Supplement: Supplementary file 2 — Supplementary Material 2: Supplementary Table 2. Occlusion analysis for the three miniplates configurations [file 12903_2026_8487_MOESM2_ESM.docx]

**Supplementary Data**

Neurosensory functional agreement between the fracture-affected side and the contralateral side was analyzed using a two-tailed Intra Class Correlation Coefficient test (ICC)^(1)^. A key to apprehending the outcome values of the ICC is presented as in supplementary table 1:

| ***Supplementary Table 1: ICC Key.*** |
| --- |
| ***<0.5 Poor agreement.*** |
| ***0.5 to <0.75 Moderate agreement.*** |
| ***0.75 to <0.9 Good agreement.*** |
| ***0.9 - 1.0 Excellent agreement.*** |

1. Han X. On Statistical Measures for Data Quality Evaluation. Journal of Geographic Information System. 2020;12:178-87. <https://doi.org/10.4236/jgis.2020.123011>.
