# Supplementary figures and images for: Impact of three miniplate configurations on mental nerve integrity in parasymphyseal mandibular fractures: a blinded randomized trial
Source: BMC Oral Health. 2026 May 7;26:859. doi: 10.1186/s12903-026-08487-0 (PMC13173844; doi:10.1186/s12903-026-08487-0)

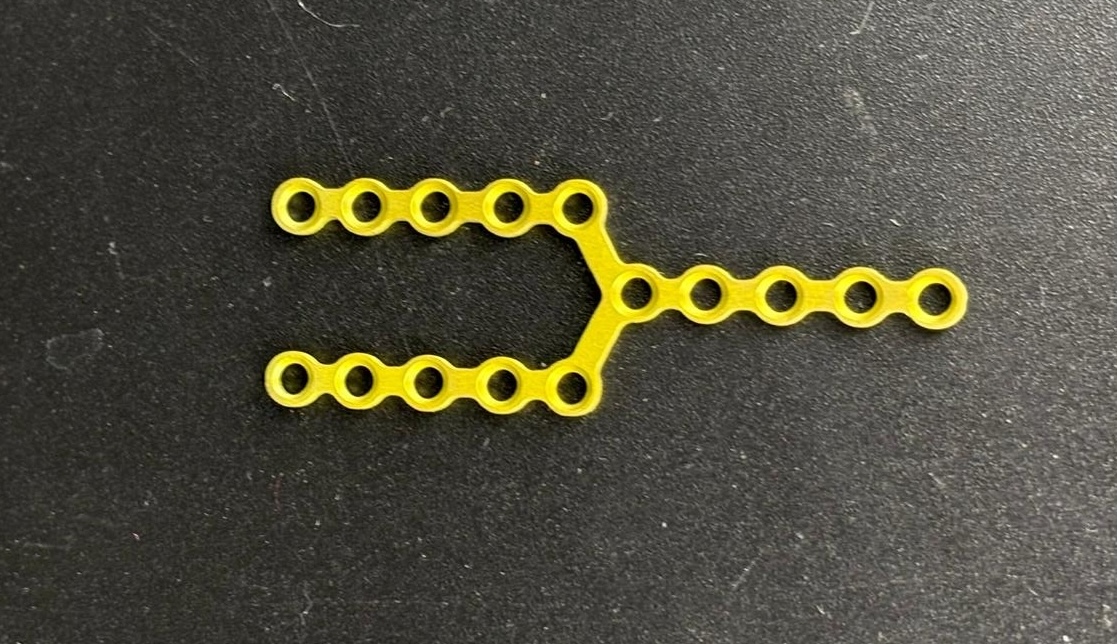

Supplement: Supplementary file 3 — Supplementary Material 3: Supplementary Figure 1. Photographic Illustration of the Twin-Fork miniplate [file 12903_2026_8487_MOESM3_ESM.jpg]

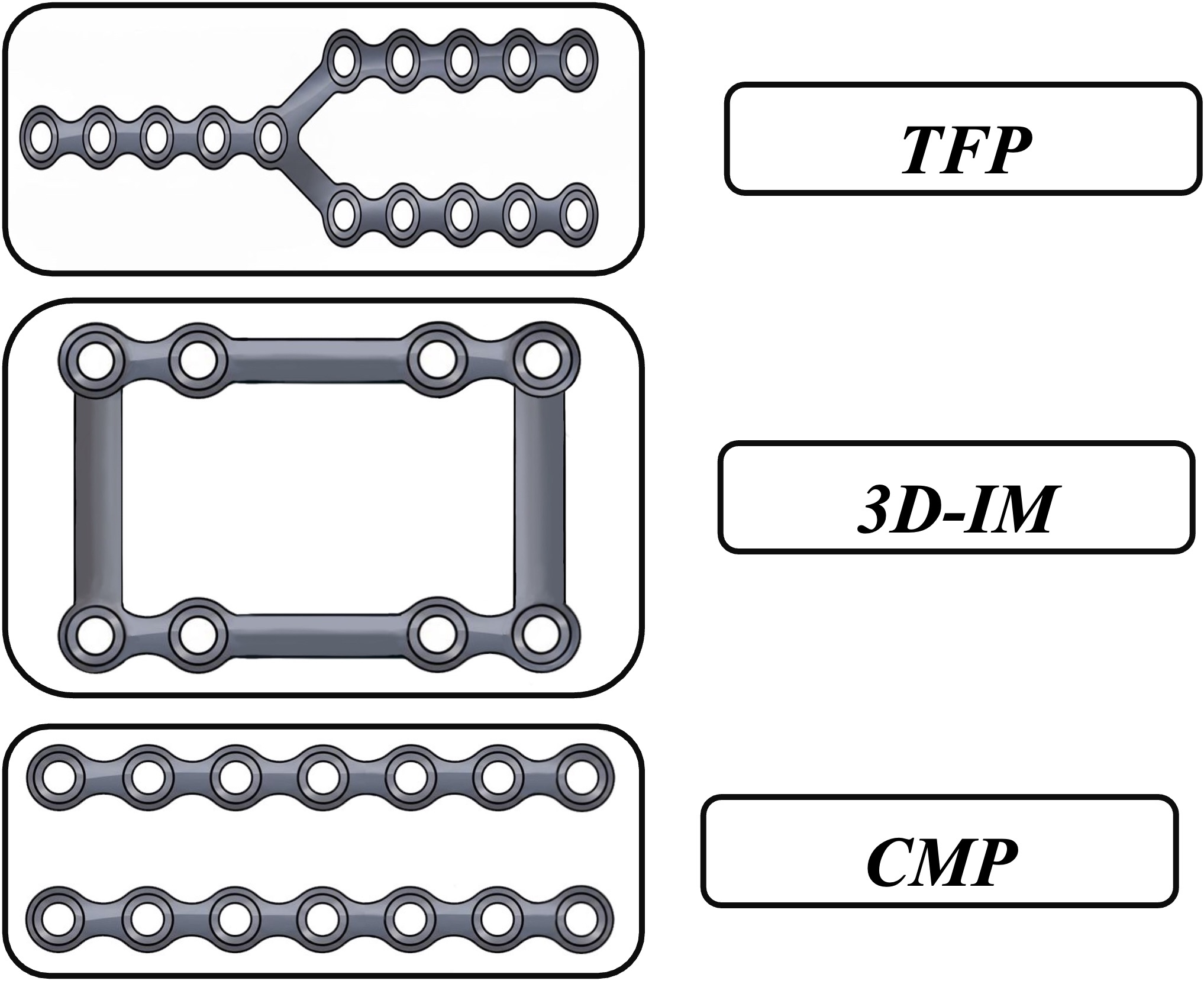

Supplement: Supplementary file 4 — Supplementary Material 4: Supplementary Figure 2. Illustration of the three miniplates configurations utilized in this study [file 12903_2026_8487_MOESM4_ESM.jpeg]
